# Health State Utility Associated with Parenteral Nutrition Requirement in Patients with Short Bowel Syndrome and Intestinal Failure in Korea: A Vignette-Based Approach

**DOI:** 10.3390/nu17223551

**Published:** 2025-11-13

**Authors:** Hyewon Sim, Jin Soo Moon, Young Suk Park, Eunji Heo, Yoon Soo Chun, Songhwa Choi, Hyemin Ku, Jae Hee Chung

**Affiliations:** 1 NDnex, 462, Bongeunsa-ro, Gangnam-gu, Seoul 16154, Republic of Korea; hw.sim@ndnex.com; 2 Department of Pediatrics, Seoul National University College of Medicine, 101 Daehak-ro, Jongno-Gu, 110-769, Seoul 03080, Republic of Korea; mjschj@snu.ac.kr; 3 Department of Surgery, Seoul National University Bundang Hospital, Seongnam 13620, Republic of Korea; yspark@snubh.org; 4 Department of Industrial Pharmaceutical Science, Ewha Womans University, Seoul 03760, Republic of Korea; eunice.heo7@gmail.com; 5 Market Access, Takeda Pharmaceuticals Korea Co., Ltd., Seoul 05551, Republic of Korea; yoon-soo.chun@takeda.com; 6 Medical Affairs, Takeda Pharmaceuticals Korea Co., Ltd., Seoul 05551, Republic of Korea; songhwa.choi@takeda.com; 7 Department of Surgery, Seoul St. Mary’s Hospital, College of Medicine, The Catholic University of Korea, Seoul 06591, Republic of Korea

**Keywords:** short bowel syndrome, intestinal failure, parenteral nutrition, health-related quality of life, utility weights

## Abstract

**Background/Objectives:** Short bowel syndrome (SBS) is the leading cause of intestinal failure (IF) that often requires long-term parenteral nutrition (PN). Extended PN results in severe complications and reduced quality of life (QoL). This study aimed to evaluate the QoL utility weights associated with PN duration using vignettes. **Methods:** We developed detailed scenarios and descriptions to represent eight hypothetical health states, reflecting variations in PN frequency in both pediatric and adult patients. A cross-sectional survey was conducted among 359 Korean adults (aged 19–59 years) from the general population, assigned to evaluate adult (n = 179) or pediatric (n = 180) vignette groups. Health utility was measured using the EuroQol 5-Dimension (EQ-5D), visual analog scale (VAS), and time trade-off (TTO) methods. Multivariable regression analysis using a mixed-effects model was employed to manage repeated measures and control for sociodemographic variables. **Results:** Utility scores measured using the EQ-5D, VAS, and TTO were negatively correlated with increasing PN days in both adult and pediatric patients with SBS-IF. The highest mean utility values were “0 days on PN” (adults: EQ-5D 0.808, VAS 0.689, TTO 0.874; pediatric: EQ-5D 0.804, VAS 0.680, TTO 0.883), while the lowest were “7 days on PN” (adults: EQ-5D 0.117, VAS 0.180, TTO 0.272; pediatric: EQ-5D 0.070, VAS 0.178, TTO 0.291). These trends remained significant after covariate adjustment (*p* < 0.001). **Conclusions:** The study revealed a steady decline in utility values with an increasing number of PN days. These findings highlight the importance of enhancing the QoL in patients with SBS-IF by supporting intestinal adaptation and reducing PN dependency.

## 1. Introduction

Short bowel syndrome (SBS) is anatomically defined as a residual small intestine length of 150–200 cm or less (excluding the duodenum), which is less than half the typical small intestine length in adults [1]. This condition usually results from the surgical removal of a significant portion of the small intestine, a procedure often performed to eliminate abnormal segments caused by conditions such as mesenteric ischemia, Crohn’s disease, or radiation enteritis in adults [2]. In pediatric cases, SBS often results from necrotizing enterocolitis in premature infants or other conditions, including volvulus or gastroschisis; however, some children are born with a shortened bowel [3].

The prevalence of SBS is low and uncertain, with reported rates ranging from 0.4 to 40 cases per million population [4,5]. Epidemiological data on SBS remain limited among Asian populations, including South Korean. In addition, SBS has been reported to occur more frequently in females than males (approximately 60–70% vs. 30–40%, respectively) [6,7]. Although SBS is generally considered a rare disease, it represents a clinically significant condition that has attracted substantial research attention, with approximately 7000 studies published globally. The occurrence of intestinal failure (IF) also varies considerably across countries, ranging from approximately 5 to 80 cases per million people [8], reflecting differences in disease etiology, healthcare systems, and population characteristics. Furthermore, SBS is recognized as one of the leading causes of IF, underscoring its critical impact on patient morbidity, mortality, and healthcare resources.

Intestinal failure (IF) can result from SBS, a condition in which the small intestine has an insufficient capacity to properly absorb macronutrients, water, and electrolytes, thereby requiring personalized medical and nutritional interventions for survival and development [2]. Patients with IF due to SBS usually require long-term, and in some cases, lifelong intravenous nutritional support [9].

Improvements in parenteral nutrition (PN) techniques have led to better outcomes in neonates, children, and adults; however, this method of nutritional support does not enhance the functional ability of the remaining bowel [10]. Additionally, PN is associated with various complications, such as those related to central venous access, including catheter-related infections, venous thrombosis, and eventual loss of venous access, as well as metabolic complications affecting the liver, kidneys, and bones [1]. Complications associated with PN not only lower health-related quality of life (HRQoL) but also increase mortality risk, underscoring the importance of reducing reliance on PN [2,11].

In addition, patients with PN dependence experience physical, psychological, and time-related challenges that complicate everyday tasks such as work, school, and travel [11]. Nocturnal PN administration can result in sleep disturbances and severe fatigue caused by noise, as well as the need for frequent nighttime urination and ongoing connection during sleep [12,13,14,15]. These issues indicate that PN dependence is the main challenge for individuals with SBS-IF, which is closely associated with a lower QoL in this patient population.

A previous study conducted in the United Kingdom evaluated utility weights based on PN days in patients with SBS-IF [16]. The study revealed the significant impact of PN frequency on the QoL among patients with SBS-IF. However, to date, no studies have assessed the QoL in Korean patients with SBS in relation to PN dependency days using preference elicitation methods such as the EuroQol 5-Dimension (EQ-5D) or time trade-off (TTO). The scarcity of research in this area highlights the limited understanding of QoL among patients with SBS in South Korea, emphasizing the need for region-specific studies to assess the economic value of new treatments and inform healthcare policy and decision-making.

Given the rarity and clinical complexity of chronic SBS-IF in South Korea, obtaining direct utility data from patients is practically difficult. Therefore, to ensure methodological feasibility and generalizability, this study adopted a general population-based valuation approach. We aimed to estimate the utility weights associated with varying durations of PN dependency in adult and pediatric patients with chronic SBS-IF, using detailed health-state vignettes, as perceived by the general population of South Korea [17].

## 2. Methods

### 2.1. Sample and Procedures

The rarity of SBS-IF in South Korea precluded recruiting a sufficient number of patients for direct utility elicitation. According to the Health Insurance Review and Assessment Service (HIRA) disease statistics, only 239 patients were treated in 2024 under the diagnostic code K91.2 (postsurgical malabsorption, not elsewhere classified). Assuming that approximately 50% of these patients require long-term parenteral nutrition and 50–75% of them have SBS [18], the estimated number of patients with chronic SBS-IF in Korea would be approximately 60–100 individuals.

Considering this limited patient population, we adopted a general-population valuation using health-state vignettes, which is a scientifically recognized and practically appropriate alternative when direct patient-based valuation is not feasible. This approach also aligns with the societal perspective recommended in health technology assessments (HTAs) and economic evaluations, enabling utility weights that reflect public preferences [19].

The sample size for the survey groups was calculated based on a previous study in which the standard deviation of the utility weights across the eight health states for patients with SBS-IF was estimated with a median value of 0.29 [16]. A sample size of 130 participants was calculated using an alpha level of 0.05 (two-sided) and a 5% margin of error. To address possible nonresponse or missing data, a 15% dropout rate was considered, resulting in a target sample size of 150 participants per group. A total of 360 participants were recruited: two groups of 180 each for the adult and pediatric HRQoL surveys, and a pilot survey of 30 participants per group. The pilot survey was conducted prior to the main one to confirm the clarity and feasibility of the questionnaire. Since no revisions were required, pilot and main survey data were combined for analysis.

Survey respondents were recruited from the general Korean population through stratified sampling based on age and sex. Individuals aged 19–59 years who voluntarily agreed to participate after being informed of the study’s objectives and methodology were included. The survey was conducted using a gang survey method, whereby participants were collected at a particular site and surveyed simultaneously [20]. Participants were categorized into two groups: those evaluating the HRQoL in adult patients with SBS-IF (hereafter referred to as the adult vignette group) and those evaluating pediatric patients (hereafter referred to as the pediatric vignette group). Each group completed their respective surveys in a single session. To address the limited knowledge of the participants regarding the health states investigated in this study, we provided descriptions and photographs that demonstrated the characteristics of adult and pediatric patients with SBS-IF in conjunction with a survey questionnaire. Participants were instructed to imagine themselves as having the health condition outlined in the scenario and answer survey questions based on that assumption, rather than their actual health situation. This approach minimized potential biases related to pre-existing diseases or diagnoses.

The Public Institutional Bioethics Committee designated by the Ministry of Health and Welfare of South Korea approved the study protocol, which included the study design, interview questionnaire, and informed consent form (P01-202401-01-056). All the participants provided written informed consent.

### 2.2. Health States Development

Health state scenarios were developed based on semi-structured, in-depth interviews with nine individuals (four pediatric and five adult patients) with SBS-IF who were recruited from three tertiary institutions in South Korea. The number of patient interviews (n = 9) was determined based on the concept of data saturation, which refers to the point in qualitative research when no new information or themes emerge from additional interviews. According to a systematic review by Hennink et al. (2022), saturation is typically reached after approximately 9–17 individual interviews or 4–8 focus group discussions [21]. Considering the low prevalence of SBS-IF in South Korea (60–100 patients nationwide), we determined that a sample of nine individuals, representing approximately 10% of the population, was considered methodologically and practically sufficient to achieve data saturation. The patient interview studies were independently approved by the institutional review boards of the three participating institutions (IRB Nos. H-2211-122-1380, B-2211-795-303, and KC23QSSI0069). Written informed consent was obtained from all participants or their caregivers prior to participation.

The interviews explored the patient experiences with SBS-IF, focusing on PN dependence, physical and emotional functioning, and social aspects of daily life. For pediatric cases, caregivers participated directly and provided detailed information regarding their child’s condition and PN-related experiences. During the sessions, patients or caregivers were presented with draft health state scenarios based on a previous study by Ballinger et al., which evaluated QoL in patients with SBS-IF according to the number of PN days [16]. Each health state scenario was structured to include multiple components, including condition, symptoms, treatments, mobility, self-care, usual activities, pain/discomfort, and anxiety/depression, based on the number of PN days). Participants reviewed the scenarios and provided feedback on their accuracy and clarity, which was used as an informal cognitive-debriefing process to refine phrasing and ensure that the vignettes were realistic, comprehensible, and conceptually valid for lay respondents.

The interview results did not yield significant modifications to the proposed scenarios. However, several minor adjustments were made to improve clarity and contextual relevance. The revised scenarios were reviewed by three clinical experts to verify medical accuracy, internal consistency, and appropriateness for the Korean clinical context. The experts’ inputs were incorporated into the final survey questionnaire and scenarios.

These validated health-state scenarios were then adapted into standardized vignettes, which served as concise, lay-friendly descriptions of SBS-IF health states for use in the general population survey (Table S1).

This multi-step development process comprising patient interviews, expert validation, and iterative refinement, ensured that the final vignettes were clinically relevant, culturally appropriate, and suitable for use in the general population survey. Each vignette included multiple domains (condition, symptoms, treatments, mobility, self-care, usual activities, pain/discomfort, and anxiety/depression) structured according to PN dependency days. Detailed examples of the finalized vignettes are provided in Supplementary Table S2.

### 2.3. Health State Valuation

HRQoL was represented by utility weights, which are quantitative single values, typically ranging from zero (death) to one (perfect health) [22]. Preference is a broader concept that encompasses utility, and various valuation methods have been used to quantify preferences for different health conditions [23]. This study used three established tools to determine utility weights: EQ-5D-5L, Visual analogue scale (VAS) and TTO. These tools were selected based on their proven reliability and validity in measuring HRQoL in population-based studies. These tools were employed to assign preference-based scores to a range of health states, with higher scores indicating preferred health conditions [24].

#### 2.3.1. EQ-5D-5L

The EQ-5D-5L is a multi-attribute utility instrument developed by the EuroQol Group that serves as a standardized measure of HRQoL. It comprises five dimensions: mobility, self-care, usual activities, pain/discomfort, and anxiety/depression. Each dimension has five levels of severity: no, slight, moderate, severe, and extreme problems [25]. The utility value (EQ-5D-5L score) was calculated using the validated Korean version of the EQ-5D-5L [26].

#### 2.3.2. VAS

The VAS uses a vertical scale to record individual subjective assessments of health status. The scale ranges from 0 to 100, with 0 representing death and 100 representing perfect health [27]. To ensure comparability with other tools, raw scores were normalized to a range between 0 and 1 [28].

#### 2.3.3. TTO

The TTO is a choice-based method used to evaluate preferences for specific health states [29]. In this approach, respondents were presented with the option of either living with a specific health condition for a fixed period (t) before death or living in perfect health for a shorter period (x) before death. The utility value was calculated as the ratio of x to t [30].

The selection of a fixed time frame in TTO tasks has been discussed extensively in the literature. Typically, the time horizon is set to either 10 or 20 years, or less frequently, determined according to actuarial life expectancy [31]. Van Nooten et al. noted that when respondents are asked to conceptualize life expectancy or age at death, subjective interpretations may bias valuation outcomes [32]. Therefore, most studies use a fixed time horizon that is independent of age or life expectancy to ensure consistency and comparability.

In previous studies measuring utilities among patients with SBS or IF, both 10-year [16] and 20-year [33] time horizons have been employed. Since there is no universally agreed-upon duration for the TTO time horizon, this study adopted a 20-year period to reflect the chronic and lifelong nature of SBS-IF. Because this study included both adult and pediatric vignettes, a 10-year horizon was considered insufficient to capture the long-term disease burden and developmental trajectory of pediatric patients. To ensure methodological consistency and comparability between the two respondent groups, the same 20-year horizon was applied to both adult and pediatric scenarios.

This decision also considered behavioral considerations. Although HRQoL and time horizons are generally regarded as independent, previous research suggests that shorter horizons may yield higher utility values, because respondents tend to be less willing to trade off remaining life years, a tendency attributed to loss aversion [32]. Therefore, a 20-year time horizon was selected to balance respondent comprehension with the minimization of excessive loss-aversion effects.

While absolute utility values may vary slightly depending on the selected duration, prior evidence shows that relative differences among health states remain stable across TTO designs [31]. Consequently, the use of a 20-year horizon in this study is unlikely to have materially impacted the interpretation of relative QoL differences across PN days.

### 2.4. Statistical Analysis

The main survey was conducted without modifying the research plan or questionnaire from the pilot survey, resulting in all participants from both surveys being included in the analysis sets for the adult and pediatric vignette groups. All analyses were conducted separately for each group.

Baseline characteristics of the study population, including demographic and health-related factors, were evaluated. Continuous variables are reported as means ± standard deviations (SD), while categorical variables are reported as frequencies (n) and percentages (%). The utility values derived from the EQ-5D-5L, VAS, and TTO for different health state scenarios, based on the number of PN days in adult and pediatric patients with SBS-IF, were compared using mean values with SD.

A multivariable regression model was used to evaluate the relationship between PN days and utility values, with demographic variables as covariates. A mixed-effects model was applied to account for the correlation between repeated measures because the participants responded repeatedly to questions in each scenario. This model allowed the inclusion of both fixed and random effects, making it suitable for analyzing longitudinal data with repeated measurements. The model accommodated unbalanced data patterns and utilized all available observations, providing a robust analysis of the relationship between PN days and utility values, while accounting for individual variability [34]. By considering the correlation between repeated measures, this approach prevented the underestimation of standard errors that could occur otherwise [35]. Data coding, cleaning, and analysis were performed using the SAS version 9.4 (SAS Institute Inc., Cary, NC, USA).

## 3. Results

### 3.1. Survey Participants

**Figure 1:**
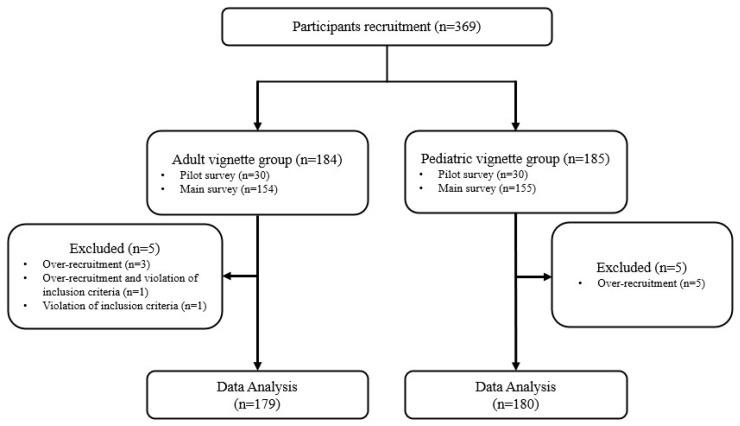
Flow chart of survey participation.

A total of 369 participants were recruited for the study, of whom 184 were assigned to the adult vignette group and 185 to pediatric vignette group. Following recruitment, there were five dropouts from each group due to violation of the inclusion criteria or exceeding the planned number of participants. The final analysis included 179 participants in the adult vignette group and 180 in pediatric vignette group (Figure 1).

The study participants were evenly distributed across age groups in both the adult and pediatric vignette groups, with a slightly higher proportion of females (57.1%, 56.8%) than males (40.2%, 40.5%) included in both groups. Approximately 2.8% of the participants in the adult vignette group and 1.7% in the pediatric vignette group reported a personal, familial, or acquaintance history of intestinal disease. One participant from the pediatric vignette group reported a known case of SBS. In terms of self-rated current health status, the majority of the study participants reported being in good health. However, 15 participants (8.4%) in the adult vignette group and 10 (5.4%) in pediatric vignette group described their health as poor. The mean HRQoL, as measured by the EQ-5D-5L, was 0.859 in the adult vignette group and 0.860 in pediatric vignette group respondents. Notably, no respondents scored at level 5 (“unable to walk,” “unable to wash or dress myself,” “unable to perform daily activities,” “have extreme pain or discomfort,” or “extremely anxious or depressed”) in any domain (Table 1).

### 3.2. Health State Utilities

**Figure 2:**
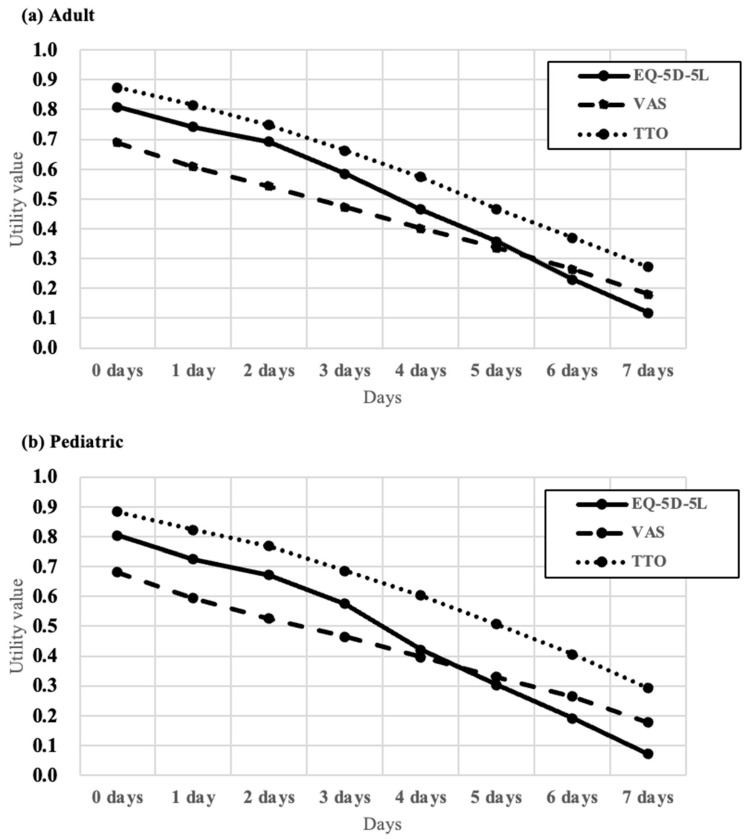
Mean utility values derived from EQ-5D-5L, VAS and TTO. (**a**) Adult vignettes, (**b**) Pediatric vignettes. EQ-5D-5L: 5-level EQ-5D; VAS: visual analogue scale; TTO: time trade-off.

The mean utility values, measured by the EQ-5D-5L, VAS, and TTO with a duration of 20 years, declined progressively as the number of PN days per week increased in both the adult and pediatric vignette groups. In adult patients, the mean utility values for 0–7 PN days per week were as follows: EQ-5D-5L: 0.808, 0.742, 0.691, 0.584, 0.464, 0.357, 0.230, and 0.117, respectively; VAS: 0.689, 0.607, 0.543, 0.472, 0.400, 0.336, 0.263, and 0.180; and TTO values were higher than the other instrument values across all scenarios at 0.874, 0.814, 0.748, 0.663, 0.574, 0.467, 0.369, and 0.272. Similar patterns of utility value change were observed in pediatric patients: EQ-5D-5L: 0.804, 0.724, 0.672, 0.575, 0.421, 0.302, 0.191, and 0.070; VAS values showed a corresponding trend with means of 0.680, 0.593, 0.525, 0.464, 0.397, 0.330, 0.264, and 0.178; and TTO values were again the highest among all instrument values at 0.883, 0.822, 0.767, 0.686, 0.603, 0.506, 0.406, and 0.291 for 0–7 PN days per week (Table 2, Figure 2).

A detailed analysis of the EQ-5D-5L domains showed that the proportion of respondents reporting higher levels of severity increased as the number of PN days per week increased across all domains in both adult and pediatric populations. The incline was notably steeper in the domains of usual activities, pain/discomfort, and anxiety/depression than in mobility and self-care (Figure S1).

A mixed model analysis, adjusted for participant demographics and repeated measures, revealed a significant decline in utility values with increasing PN days in both adult and pediatric patients (all *p* < 0.0001). The difference in mean utility values between the consecutive health states based on the number of PN days per week, as measured by the EQ-5D, ranged from 0.05 to 0.13 in adults and from 0.05 to 0.15 in pediatric patients. Similarly, the decrease in utility per additional day of PN, as measured by the VAS, ranged from 0.06 to 0.08 in adults and from 0.06 to 0.09 in pediatric patients. When measured by the TTO, the decrease was 0.06 to 0.11 in both adult and pediatric populations. These difference values were >0.036, which was the minimum level of change for a clinically meaningful change in the EQ-5D index scores, as identified in previous studies (Table 3, Tables S3 and S4) [26,36]. The goodness-of-fit indices for the mixed-effects model supported its adequacy in both the adult and pediatric vignette groups. For the adult group, −2 Log Likelihood (−2LL) = −3251.9, Akaike Information Criterion (AIC) = −3147.9, corrected AIC (AICc) = −3143.9, and Bayesian Information Criterion (BIC) = −2982.1; for the pediatric group, −2LL = −3255.7, AIC = −3151.7, AICc = −3147.7, and BIC = −2985.6 (both *p* < 0.001 vs. the null model). These results indicate that the mixed-effects model appropriately accounted for the repeated-measures structure of the data and demonstrated robust model fit across groups.

To assess inter-subject variability across PN days, a mixed-effects model with random intercepts and a PN day-specific heteroscedasticity covariance structure was applied. The estimated between-subject standard deviation was 0.07 in the adult group and 0.08 in pediatric group, indicating comparable variability between groups. Within-subject variance across PN days ranged from 0.001 to 0.022 in adults and 0.002 to 0.014 in pediatrics. The corresponding intra-class correlation coefficients (ICCs) were 0.18–0.82 (median ≈ 0.32) and 0.31–0.72 (median ≈ 0.42), respectively, suggesting moderate consistency of individual responses across PN days. These results support the suitability of the mixed-effects model in accounting for repeated measures over PN day progression (Table S5).

## 4. Discussion

This population-based survey assessed the QoL in patients with SBS-IF in South Korea by estimating utility values based on the number of PN days per week. The study findings revealed that utility values measured using the EQ-5D-5L, VAS, and TTO steadily declined as the number of PN days per week increased in both adult and pediatric patient groups in Korea. These results highlight the considerable treatment burden and impaired HRQoL associated with PN dependency in patients with SBS-IF. The consistent trend observed across instruments underscores the robustness of the findings.

The QoL in patients with SBS-IF, represented by the EQ-5D-5L results in this study, was generally lower than that observed in the general population and fell within the range reported for other chronic diseases or organ failure. For example, patients with stable chronic heart failure reported a high utility value of 0.871, whereas those with SBS not requiring PN in this study had slightly lower scores (0.808 in adults and 0.804 in children). Among those requiring PN support, utility values declined substantially with increasing PN days, indicating a considerable disease burden. The magnitude of these utility losses was comparable to that observed in other serious chronic conditions such as end-stage renal disease or very severe COPD) [37,38]. These comparisons are provided solely to contextualize the relative extent of HRQoL impairment in SBS-IF, and should be interpreted with caution given the distinct clinical characteristics across diseases.

The decline in the QoL among patients with SBS-IF associated with an increased PN duration may be attributed to a higher risk of complications and infections, potentially resulting in more frequent hospitalizations and increased mortality [1,39,40]. Furthermore, prolonged PN use adversely affects sleep quality and presents substantial challenges to maintaining social interactions [11,12]. This impairment in QoL due to long-term treatment burden could be better understood in relation to other diseases with similar management strategies, such as chronic kidney disease. A study evaluating QoL in patients with end-stage renal disease undergoing repeated hemodialysis showed that an increase in the weekly frequency of hemodialysis was associated with a decrease in HRQoL, mirroring our study findings for patients with SBS receiving PN [41]. This finding suggests that repetitive, time-consuming, and invasive procedures have a negative impact on patients’ QoL and implies that improvements in QoL are expected in SBS-IF patients who achieve a reduction in PN days [16,42,43,44,45,46,47].

The utility weights for each health state were higher when using the TTO instrument than when using the other instruments for both adult and pediatric patients. This phenomenon may be attributed to the nature of the TTO, which involves a choice between the health state and death, leading individuals to assign higher utility to the health state to avoid the extreme alternative of death [24]. Additionally, utility weights derived from the EQ-5D-5L and VAS scores were lower in pediatric than in adult patients, while those measured by TTO were higher in pediatric patients. Given the inherently longer life expectancy and greater developmental potential of children, proxies may exhibit a natural reluctance to consider trading life years during TTO evaluations. This tendency potentially resulted in greater emphasis on the preservation of life, leading to higher utility values assigned in TTO-based assessments [48].

The utility values obtained through the indirect EQ-5D-5L instrument were consistently lower across all health states compared to those measured by the TTO, aligning with the general consensus in the field [49] and previous research findings [16]. However, when comparing utility values obtained from the EQ-5D-5L with those measured by the VAS, the EQ-5D-5L values were higher than the VAS values in conditions with less frequent PN days. This discrepancy likely arises from the ceiling effect, which is a known limitation of the EQ-5D tool. Although the EQ-5D-5L is assessed at five levels, thereby increasing descriptive richness and mitigating the ceiling effect compared to its predecessor, the EQ-5D-3L [50,51], it does not entirely eliminate this limitation [52]. Consequently, health states with fewer PN days may exhibit higher utility weights due to the ceiling effect.

For a robust vignette approach, the construction of vignettes that can accurately and sensitively capture health state preferences are essential to ensure a reflection of patient typical experiences and common attributes [17]. To ensure validity and sensitivity in the vignette approach, the vignettes used in this study were developed based on extensive interviews with patients diagnosed with SBS and reviewed by clinical experts. This approach conforms to the suggestion of the Decision Support Unit in Technical Support Document 11, published by NICE [53]. Moreover, the sample of 360 respondents was stratified to reflect the demographic composition of the Korean general population, thereby representing collective societal preferences. By bridging clinical experience and social valuation, this design strengthens the external validity of the utility estimates. Nevertheless, it should be acknowledged that health condition vignettes may have inherent limitations in terms of fully encapsulating the breadth of patient lived experiences, and future research incorporating patient-reported data may further enhance the realism of such valuations.

Our study results are consistent with those of a previous study conducted in the UK [16], which also reported declining utility values with increasing PN days. However, our study extends this evidence in keyways. While the UK study focused solely on adults, we measured utility weights in both adult and pediatric patients, enabling assessment of PN’s impact across the life course. In addition to direct valuation methods VAS and TTO used in the UK study, we employed the EQ-5D-5L as a primary indirect instrument. Because EQ-5D–based utilities are widely used in HTA and reimbursement processes, our findings provide evidence that can inform cost-utility evaluations and support broader understanding of the social value associated with PN-related health states. We also modeled PN frequency as a categorical variable, allowing direct comparisons between consecutive PN-day states (e.g., 0 vs. 1 day, 1 vs. 2 days). This approach identified statistically significant differences between adjacent PN states, offering a more detailed understanding of how incremental increases in PN dependency influence perceived quality of life. Together, these findings contribute novel, age-inclusive utility data that complement and extend existing UK-based evidence.

The prognosis of SBS-IF has improved substantially with the establishment of specialized intestinal failure programs and multidisciplinary care. Recent reports from expert centers indicate a 5-year survival rate of approximately 81.9% in patients receiving comprehensive intestinal rehabilitation [54,55]. However, life expectancy remains lower than that of the general population, and long-term PN dependence continues to be associated with significant risks, including PN-associated liver disease and metabolic complications [56]. Optimizing PN management protocols and expanding access to advanced intestinal rehabilitation programs remain essential for improving both survival and QoL in this population. Recent advances in intestinal rehabilitation and emerging therapeutic modalities have shown promise in further improving the long-term outcomes of patients with SBS-IF. Pharmacological interventions that promote intestinal adaptation, such as glucagon-like peptide-2 (GLP-2) analogs (e.g., teduglutide) and novel surgical techniques, including bowel lengthening and intestinal valve transplantation, have demonstrated potential to reduce PN dependency and enhance health-related QoL [42,43,44,45,57,58]. Continued investigation into these approaches may yield meaningful clinical and economic benefits by alleviating the chronic treatment burden associated with SBS-IF.

While vignette-based valuations have inherent limitations, this study makes a novel contribution by providing the first utility-based assessment of HRQoL in patients with SBS-IF in relation to PN dependency days in South Korea. Using a representative sample of the Korean general population, our study offers valuable insights into the impact of PN dependency on QoL in both pediatric and adult patients with SBS-IF, thereby providing crucial information for healthcare policy and decision-making. Recently, SBS and IF were included in the International Classification of Diseases, 11th Revision, which has initiated discussions on the integration of these codes into the national classification system in South Korea [59]. This development will facilitate more accurate epidemiological assessments and improve research methodologies and diagnostic criteria, thereby strengthening the evidence base for future investigations of SBS and IF.

## 5. Conclusions

This study estimated utility weights for PN-associated health states in patients with SBS using a representative sample of the general Korean population and a robust vignette development approach. These findings revealed a progressive decline in the utility values as the number of PN days increased, underscoring the critical need to enhance the QoL in both adult and pediatric patients with SBS-IF. These results highlighted the importance of promoting intestinal adaptation and minimizing the frequency of PN to improve patient outcomes and quality of life.

## Figures and Tables

**Table 1:** General characteristics of survey participants.

Characteristics	Adult Vignettes (n = 179)	Pediatric Vignettes (n = 180)
Age (years), n (%)		
19–29	47 (26.3%)	48 (26.7%)
30–39	44 (24.6%)	44 (24.4%)
40–49	47 (26.3%)	49 (27.2%)
50–59	41 (22.9%)	39 (21.7%)
Sex, n (%)		
Male	74 (41.3%)	75 (41.7%)
Female	105 (58.7%)	105 (58.3%)
Education, n (%)		
High school graduate or lower	11 (6.1%)	6 (3.3%)
University attendance or higher	168 (93.9%)	174 (96.7%)
Monthly household income (KRW), n (%)		
≤5 million	56 (31.3%)	57 (31.7%)
>5 million	123 (68.7%)	123 (68.3%)
Employment status, n (%)		
Employed	118 (65.9%)	138 (76.7%)
Student/homemaker	59 (33.0%)	39 (21.7%)
Unemployed	2 (1.1%)	3 (1.7%)
Health insurance, n (%)		
NHI district subscriber	51 (28.5%)	38 (21.1%)
NHI employee subscriber	119 (66.5%)	134 (74.4%)
Medical aid	6 (3.4%)	3 (1.7%)
Don’t know	3 (1.7%)	5 (2.8%)
Disease (self or family/acquaintances) *, n (%)		
Short Bowel Syndrome	0 (0%)	1 (0.6%)
Other intestinal malabsorption disorders	5 (2.8%)	2 (1.1%)
None	174 (97.2%)	177 (98.3%)
Current health status, n (%)		
Normal or improved	164 (91.6%)	170 (94.4%)
Poor or declined	15 (8.4%)	10 (5.6%)
EQ-5D-5L, mean (SD)	0.86 ± 0.05	0.86 ± 0.05
VAS, mean (SD)	0.82 ± 0.11	0.82 ± 0.13

* Duplicate responses are possible. KRW: South Korean Won; NHI: National Health Insurance; EQ-5D-5L: 5-level EQ-5D; VAS: visual analogue scale.

**Table 2:** Mean utility values of the number of PN days per week derived from EQ-5D-5L, VAS and TTO.

PN Days	Adult Vignettes			Pediatric Vignettes		
	EQ-5D-5L	VAS	TTO	EQ-5D-5L	VAS	TTO
0 days	0.808 (0.062)	0.689 (0.130)	0.874 (0.111)	0.804 (0.059)	0.680 (0.146)	0.883 (0.120)
1 day	0.742 (0.073)	0.607 (0.130)	0.814 (0.127)	0.724 (0.091)	0.593 (0.148)	0.822 (0.127)
2 days	0.691 (0.098)	0.543 (0.123)	0.748 (0.141)	0.672 (0.108)	0.525 (0.144)	0.767 (0.134)
3 days	0.584 (0.146)	0.472 (0.121)	0.663 (0.157)	0.575 (0.147)	0.464 (0.143)	0.686 (0.145)
4 days	0.464 (0.176)	0.400 (0.120)	0.574 (0.175)	0.421 (0.169)	0.397 (0.138)	0.603 (0.155)
5 days	0.357 (0.161)	0.336 (0.123)	0.467 (0.191)	0.302 (0.137)	0.330 (0.132)	0.506 (0.174)
6 days	0.230 (0.172)	0.263 (0.121)	0.369 (0.201)	0.191 (0.144)	0.264 (0.128)	0.406 (0.184)
7 days	0.117 (0.170)	0.180 (0.119)	0.272 (0.209)	0.070 (0.134)	0.178 (0.119)	0.291 (0.190)

Values are presented as mean (SD). PN: parenteral nutrition; EQ-5D-5L: 5-level EQ-5D; VAS: visual analogue scale; TTO: time trade-off.

**Table 3:** Result of mixed effects model repeat measures (MMRM) analysis: EQ-5D-5L.

Variable	Adult Vignettes		Pediatric Vignettes	
	Coefficient	*p*	Coefficient	*p*
Constant	0.7911	<0.0001	0.8331	<0.0001
0 days on PN (weaned off)				
1 day on PN	−0.0665	<0.0001	−0.0800	<0.0001
2 days on PN	−0.1173	<0.0001	−0.1327	<0.0001
3 days on PN	−0.2242	<0.0001	−0.2287	<0.0001
4 days on PN	−0.3442	<0.0001	−0.3834	<0.0001
5 days on PN	−0.4511	<0.0001	−0.5019	<0.0001
6 days on PN	−0.5782	<0.0001	−0.6128	<0.0001
7 days on PN	−0.6908	<0.0001	−0.7338	<0.0001
Age	0.0004	0.4325	0.0005	0.2256
Sex (Male)				
Female	−0.0015	0.8805	−0.0004	0.9634
Education (High school graduate or lower)				
University attendance or higher	−0.0016	0.9329	−0.0384	0.1096
Monthly household income (≤5 million KRW)				
>5 million KRW	0.0113	0.2606	−0.0120	0.2230
Employment status (Unemployed/Student/Homemaker)				
Employed	−0.0046	0.6599	−0.0032	0.7630
Health insurance (National Health Insurance)				
Medical aid	0.0272	0.2867	−0.0238	0.4724
Don’t know	0.0308	0.3966	−0.0017	0.9511
Disease (No)				
Yes	−0.0260	0.3375	−0.0228	0.4890
Difference of Least square means	Differences	*p*	Differences	*p*
Between PN days				
0 days vs. 1 day	0.0665	<0.0001	0.0800	<0.0001
1 day vs. 2 days	0.0508	<0.0001	0.0527	<0.0001
2 days vs. 3 days	0.1069	<0.0001	0.0960	<0.0001
3 days vs. 4 days	0.1200	<0.0001	0.1547	<0.0001
4 days vs. 5 days	0.1070	<0.0001	0.1185	<0.0001
5 days vs. 6 days	0.1271	<0.0001	0.1109	<0.0001
6 days vs. 7 days	0.1126	<0.0001	0.1210	<0.0001

EQ-5D-5L: 5-level EQ-5D; PN: parenteral nutrition; KRW: South Korean Won.

## Data Availability

The data presented in this study are available on request from the corresponding author. The data are not publicly available, due to ethical considerations.

## Supplementary Materials

The following supporting information can be downloaded at: https://www.mdpi.com/article/10.3390/nu17223551/s1, Figure S1: Proportion of responses by level of severity for EQ-5D-5L dimensions; Table S1: Description of health states; Table S2: Example of Vignette Developed from the Health State Scenarios; Table S3: Results of mixed effects model repeated measures analysis: VAS; Table S4: Results of mixed effects model repeated measures analysis: TTO; Table S5: Mixed-Effects Model Fit Statistics and Variance Components.

## Abbreviations

The following abbreviations are used in this manuscript:

−2LL	−2 Log Likelihood
AIC	Akaike Information Criterion
AICc	Corrected AIC
BIC	Bayesian Information Criterion
EQ-5D	EuroQol 5-Dimension
EQ-5D-5L	5-level EQ-5D
HRQoL	Health-related quality of life
ICCs	Intra-class correlation coefficients
IF	Intestinal failure
KRW	South Korean Won
NHI	National Health Insurance
PN	Parenteral nutrition
QoL	Quality of life
SBS	Short bowel syndrome
SD	Standard deviation
TTO	Time trade-off
VAS	Visual analog scale
