# Supplementary material for: Health State Utility Associated with Parenteral Nutrition Requirement in Patients with Short Bowel Syndrome and Intestinal Failure in Korea: A Vignette-Based Approach
Source: Nutrients. 2025 Nov 13;17(22):3551. doi: 10.3390/nu17223551 (PMC12654975; doi:10.3390/nu17223551)
Supplement: Supplementary file 1 [file nutrients-17-03551-s001.zip › nutrients-3920604-supplementary.pdf]

Supplementary Materials

Figure S1. Proportion of responses by level of severity for EQ-5D-5L dimensions

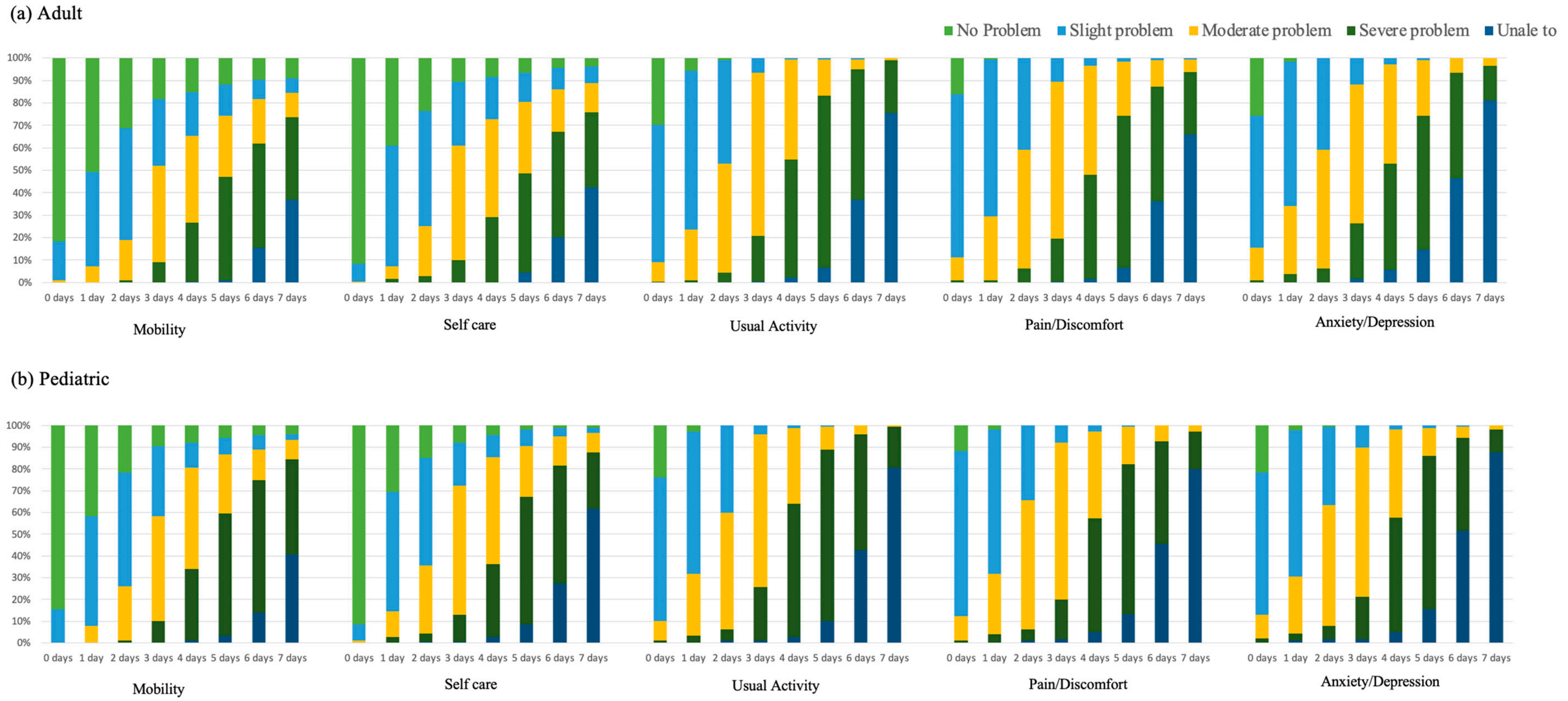

EQ-5D-5L: 5-level EQ-5D

Table S1. Description of health states

Category	Description
Condition description	• 0 days on PN: You have a condition where you need to pay attention to your fluid and nutrient intake<br>• 1-7 days on PN: You have a condition where you need to pay attention to your fluid and nutrient intake and cannot absorb nutrients normally.
Symptom condition	• 0 days on PN: You may feel dehydrated, weak and tired. You sometimes have diarrhea and suddenly need to have a bowel movement.<br>• 1-3 days on PN: (as per 0 days) and you have to limit your food and drink intake.<br>• 4-7 days on PN: (as per 1-3 days) and minor change from 'you sometimes have diarrhea' to 'you have diarrhea'
Treatment description	• 0 days on PN: You take medication to treat your diarrhea. You have a daily allowance in the amount you can drink. You have a diet that needs to be high in fat. Sometimes you need to drink a litter of a glucose-saline drink, which tastes a bit like sea water.<br>• 1-7 days on PN: You have nutrient solution through a tube in your chest for 12-16 hours [NUMBER OF DAYS] day a week. This supplements the food and drink that you eat normally.
Mobility	• 0-7 days on PN: You have no physical problems walking about.
Self-care	• 0 days on PN: You have no problems washing and dressing yourself.<br>• 1-7 days on PN: (in addition to 0 days) and you need to be cautious when washing yourself to minimize risk of infection to your tube. You need to get up in the night to urinate.
Usual activities	• 0 days on PN: Due to diarrhea and fatigue, you are sometimes limited in your usual activities.<br>• 1-3 days on PN: (in addition to 0 days) and due to having a tube you are unable to do physical exercise. Due to time for medical care, you are sometimes limited in your daily activities on your [NUMBER OF DAYS] of treatment.<br>• 4-5 days on PN: (as per 1-3 days) and minor change from 'you are sometimes limited' to 'you are limited in your daily activities (on your [NUMBER OF DAYS] of treatment)'.<br>• 6-7 days on PN: (as per 4-5 days) and it is difficult to take part in spontaneous activities.
Pain / Discomfort	• 0-7 days on PN: You have pain if you eat fibrous food.
Anxiety / Depression	• 0 days on PN: You are glad that you do not need to receive nutrients through a tube in your chest.<br>• 1-5 days on PN: You sometimes worry about getting an infection and your long-term health. You feel anxious if you need to get to the toilet quickly. You sometimes feel your treatment is a bit difficult to cope with.<br>• 6 days on PN: (as per 1-5 days) and you value having 1 day per week without having treatment.<br>• 7 days on PN: (as per 1-5 days) and you would value having 1 day per week without having treatment.

PN: parenteral nutrition.
Source: [17]

Table S2. Example of Vignette Developed from the Health State Scenarios

Weekly 1-Day Parenteral Nutrition
**You are an adult patient with short bowel syndrome accompanied by chronic intestinal failure, and you receive parenteral nutrition once per week.**<br><br>Because you cannot absorb nutrients normally, you must pay close attention to your fluid and nutrient intake. You may experience dehydration, weakness, and fatigue, and sometimes have diarrhea or an urgent need to use the toilet. You also need to restrict the types and amounts of food and beverages you consume.<br><br>For 12–16 hours on one day each week, you receive a nutrient solution through a catheter inserted in your chest. This infusion supplements the food and fluids you take orally.<br><br>**When receiving parenteral nutrition once per week:**<br>• You often need to wake up at night to urinate.<br>• You daily activities are limited on the day of parenteral nutrition infusion.<br><br>**In general**<br>• You have no physical problems with walking.<br>• You can wash and dress yourself without difficulty.<br>• You need to be cautious when washing to minimize the risk of catheter-related infection.<br>• You sometimes worry about infections and the health of your internal organs.<br>• Due to diarrhea and fatigue, your usual activities are occasionally restricted.<br>• You feel anxious when you urgently need to go to the toilet.<br>• You avoid vigorous physical exercise because of the catheter.<br>• You experience pain when eating high-fiber foods.<br>• You sometimes find it quite difficult to cope with the treatment.

Table S3. Results of mixed effects model repeated measures analysis: VAS

Variable	Adult vignettes		Pediatric vignettes	
	Coefficient	p	Coefficient	p
Constant	0.6571	<.0001	0.6698	<.0001
0 days on PN (weaned off)				
1 day on PN	-0.0819	<.0001	-0.0868	<.0001
2 days on PN	-0.1463	<.0001	-0.1549	<.0001
3 days on PN	-0.2175	<.0001	-0.2162	<.0001
4 days on PN	-0.2889	<.0001	-0.2833	<.0001
5 days on PN	-0.3532	<.0001	-0.3505	<.0001
6 days on PN	-0.4258	<.0001	-0.4163	<.0001
7 days on PN	-0.5093	<.0001	-0.5026	<.0001
Age	0.0019	0.0097	0.0012	0.1238
Sex (Male)				
Female	0.0390	0.0177	0.0323	0.0567
Education (High school graduate or lower)				
University attendance or higher	-0.0900	0.0035	-0.1011	0.0247
Monthly household income (≤5 million KRW)				
>5 million KRW	0.0221	0.1757	0.0148	0.4218
Employment status (Unemployed/Student/Homemaker)				
Employed	-0.0002	0.9895	0.0426	0.0342
Health insurance (National Health Insurance)				
Medical aid	0.0309	0.4590	-0.0878	0.1579
Don't know	0.0188	0.7504	0.0081	0.8726
Disease (No)				
Yes	0.0405	0.3583	0.0225	0.7148
**Difference of Least square means**	Differences	p	Differences	p
Between PN days				
0 days vs 1 day	0.0819	<.0001	0.0868	<.0001
1 day vs 2 days	0.0644	<.0001	0.0681	<.0001
2 days vs 3 days	0.0712	<.0001	0.0613	<.0001
3 days vs 4 days	0.0715	<.0001	0.0671	<.0001
4 days vs 5 days	0.0643	<.0001	0.0672	<.0001
5 days vs 6 days	0.0726	<.0001	0.0658	<.0001
6 days vs 7 days	0.0835	<.0001	0.0863	<.0001

VAS: visual analogue scale; PN: parenteral nutrition; KRW: South Korean Won.

Table S4. Results of mixed effects model repeated measures analysis: TTO

Variable	Adult vignettes		Pediatric vignettes	
	Coefficient	p	Coefficient	p
Constant	0.9026	<.0001	0.7877	<.0001
0 days on PN (weaned off)				
1 day on PN	-0.05999	<.0001	-0.06042	<.0001
2 days on PN	-0.1265	<.0001	-0.1157	<.0001
3 days on PN	-0.2116	<.0001	-0.1969	<.0001
4 days on PN	-0.3006	<.0001	-0.2800	<.0001
5 days on PN	-0.4076	<.0001	-0.3765	<.0001
6 days on PN	-0.5051	<.0001	-0.4769	<.0001
7 days on PN	-0.6021	<.0001	-0.5917	<.0001
Age	0.0001	0.8628	0.0018	0.0351
Sex (Male)				
Female	0.0197	0.2722	0.0366	0.0396
Education (High school graduate or lower)				
University attendance or higher	-0.0528	0.1149	0.0244	0.6021
Monthly household income (≤5 million KRW)				
>5 million KRW	0.0278	0.1209	-0.0359	0.0646
Employment status (Unemployed/Student/Homemaker)				
Employed	-0.0180	0.3412	0.0086	0.6823
Health insurance (National Health Insurance)				
Medical aid	-0.0540	0.2382	-0.1329	0.0423
Don't know	-0.1215	0.0628	0.0672	0.2071
Disease (No)				
Yes	0.0329	0.4969	0.0063	0.9224
**Difference of Least square means**	Differences	p	Differences	p
Between PN days				
0 days vs 1 day	0.0600	<.0001	0.0604	<.0001
1 day vs 2 days	0.0666	<.0001	0.0553	<.0001
2 days vs 3 days	0.0851	<.0001	0.0812	<.0001
3 days vs 4 days	0.0890	<.0001	0.0832	<.0001
4 days vs 5 days	0.1070	<.0001	0.0965	<.0001
5 days vs 6 days	0.0976	<.0001	0.1003	<.0001
6 days vs 7 days	0.0970	<.0001	0.1148	<.0001

TTO: time trade-off; PN: parenteral nutrition; KRW: South Korean Won.

Table S5. Mixed-Effects Model Fit Statistics and Variance Components

	EQ-5D-5D			VAS			TTO		
**Adult vignette groups**									
Fit Statistics									
-2 Log Likelihood		-3251.9			-5151.6			-3724.2	
AIC (Smaller is Better)		-3147.9			-5047.6			-3620.2	
AICc (Smaller is Better)		-3143.9			-5043.6			-3616.2	
BIC (Smaller is Better)		-2982.1			-4881.8			-3454.5	
Null Model Likelihood Ratio Test	DF	χ^2	p	DF	χ^2	p	DF	χ^2	p
	35	1615.7	<.0001	35	3059.95	<.0001	35	2470.37	<.0001
Intersubject Variability	Within-Subject Var	ICC	Between-Subject SD	Within-Subject Var	ICC	Between-Subject SD	Within-Subject Var	ICC	Between-Subject SD
0 days on PN	0.002812	0.6344	0.069864	0.009035	0.5649	0.108305	0.012264	0.5856	0.131643
1 day on PN	0.001096	0.8167	0.069864	0.005998	0.6617	0.108305	0.007549	0.6966	0.131643
2 days on PN	0.002398	0.6705	0.069864	0.003461	0.7722	0.108305	0.003389	0.8364	0.131643
3 days on PN	0.010533	0.3167	0.069864	0.001049	0.9179	0.108305	0.001236	0.9334	0.131643
4 days on PN	0.017994	0.2134	0.069864	0.00027	0.9775	0.108305	0.00355	0.8300	0.131643
5 days on PN	0.015697	0.2372	0.069864	0.001004	0.9211	0.108305	0.007531	0.6971	0.131643
6 days on PN	0.020889	0.1894	0.069864	0.002941	0.7996	0.108305	0.013233	0.5670	0.131643
7 days on PN	0.021953	0.1819	0.069864	0.006122	0.6571	0.108305	0.018772	0.4800	0.131643
**Pediatric vignette groups**									
Fit Statistics									
-2 Log Likelihood		-3255.7			-5101.3			-3995.9	
AIC (Smaller is Better)		-3151.7			-4997.3			-3891.9	
AICc (Smaller is Better)		-3147.7			-4993.4			-3887.9	
BIC (Smaller is Better)		-2985.6			-4831.3			-3725.8	
Null Model Likelihood Ratio Test	DF	χ^2	p	DF	χ^2	p	DF	χ^2	p

	35	1372.04	<.0001	35	3364.07	<.0001	35	2657.8	<.0001
Intersubject Variability	Within-Subject Var	ICC	Between-Subject SD	Within-Subject Var	ICC	Between-Subject SD	Within-Subject Var	ICC	Between-Subject SD
0 days on PN	0.004724	0.5779	0.080424	0.009027	0.6405	0.126807	0.011202	0.6086	0.131985
1 day on PN	0.00249	0.722	0.080424	0.00525	0.7539	0.126807	0.005009	0.7767	0.131985
2 days on PN	0.002956	0.6864	0.080424	0.002423	0.8691	0.126807	0.002934	0.8559	0.131985
3 days on PN	0.00874	0.4253	0.080424	0.000934	0.9451	0.126807	0.001147	0.9382	0.131985
4 days on PN	0.014172	0.3134	0.080424	0.000441	0.9733	0.126807	0.002468	0.8759	0.131985
5 days on PN	0.009004	0.418	0.080424	0.001526	0.9133	0.126807	0.006117	0.7401	0.131985
6 days on PN	0.011982	0.3506	0.080424	0.003295	0.83	0.126807	0.010159	0.6316	0.131985
7 days on PN	0.012801	0.3357	0.080424	0.007118	0.6932	0.126807	0.016204	0.5181	0.131985

EQ-5D-5L: 5-level EQ-5D; VAS: visual analogue scale; TTO: time trade-off; AIC: Akaike Information Criterion; AICc: corrected AIC; BIC: Bayesian Information Criterion; PN: parenteral nutrition; DF: Degrees of Freedom
